# Sensitivity of Three Impact Assessment Methodologies in Adjusting Preventive Chemotherapy Treatment Decisions for Schistosomiasis Elimination in Ondo State, Nigeria

**DOI:** 10.4269/ajtmh.24-0352

**Published:** 2025-02-18

**Authors:** Uwem F. Ekpo, Francisca O. Olamiju, Hammed O. Mogaji, Samuel N. Ovia, Olanike O. Oladipupo, Alice Y. Kehinde, Fatai O. Oyediran, Moses Aderogba, Louise K. Makau-Barasa

**Affiliations:** ^1^ Federal University of Agriculture, Abeokuta, Nigeria;; ^2^ Akwa Ibom State University, Ikot Akpaden, Nigeria;; ^3^ Mission to Safe the Helpless, Lagos, Nigeria;; ^4^ Marian University, Indianapolis, Indiana;; ^5^ Neglected Tropical Diseases Program Unit, Department of Public Health, Ondo State Ministry of Health, Akure, Nigeria;; ^6^ Neglected Tropical Diseases Program Unit, Department of Public Health, Federal Ministry of Health, Akure, Nigeria;; ^7^ Neglected Tropical Diseases Program Unit, Department of Public Health, Federal Ministry of Health, Abuja, Nigeria;; ^8^ Ending Neglected Diseases Fund, New York, New York

## Abstract

Efforts to eliminate schistosomiasis in Africa have advanced, with most countries evaluating the impact of preventive chemotherapy (PC) on disease burden. WHO has recommended eight distinct methodologies for such assessment. We, therefore, investigated the sensitivity of three prominent methodologies—sentinel, cluster, and practical, each varying in site selection, sampling approach, and data interpretation. We conducted a cross-sectional study among 2,093 children across 45 schools in Ese-Odo, Ile-Oluiji, and Irele local government areas (LGAs) of Ondo, Nigeria. Fresh stool and urine samples were processed using Kato–Katz and urine filtration techniques to estimate prevalence, which was compared with 2014 baseline estimates. Findings showed significant prevalence reductions in Ese-Odo from 1.3% (95% CI: 0.5–3.3) at baseline to 0.1% (95% CI: 0.01–0.95) at impact (d = −92.3%, *P* = 0.03) and in Ile-Oluiji from 58.0% (95% CI: 53.9–62.1) to 1.8% (95% CI: 0.9–3.3; d = −97%, *P* = 0.00). However, it increased from 3.0% (95% CI: 1.6–5.6) to 5.3% (95% CI: 3.8–7.3) in Irele (d = 66%, *P* = 0.13). Higher prevalence estimates were observed with the practical method compared with cluster and sentinel across the three LGAs: 0.3% versus 0.1% versus 0.0% in Ese-Odo, 5.8% versus 5.3% versus 5.4% in Irele, and 2.2% versus 1.8% versus 1.5% in Ile-Oluiji (all *P* >0.05). Sentinel and cluster methodologies suggest stopping PC, whereas the practical method suggests continued PC in Irele. Our findings demonstrate that practical assessment is a sensitive method for refining PC decisions.

## INTRODUCTION

Schistosomiasis or bilharziasis is a neglected tropical disease (NTD) with over 200 million people across 78 countries currently affected.^1^^–^^3^ In sub-Saharan Africa, human infections are commonly caused by trematode parasites of the genus *Schistosoma*, namely *Schistosoma hematobium* and *Schistosoma mansoni*,^1^ in marginalized urban, semiurban, and rural populations; people are exposed through surface water contact practices that harbor both the larval stages of the parasites and their intermediate snail hosts of the genera *Bulinus* and *Biomphalaria*.^1^^,^^4^ Schistosomiasis is an NTD targeted for elimination as a public health problem (EPHP) by 2030.^3^ The strategy called preventive chemotherapy (PC) involves mass administration of praziquantel drugs in schools or communities using the WHO endemicity classification system and guidelines.^5^ This strategy aims to reduce the morbidity of heavy-intensity infections in at-risk populations to below 1% across 78 countries and achieve EPHP by 2030.^3^

In the last two decades, WHO has coordinated the annual distribution of over 250 million praziquantel tablets to several endemic countries, with approximately 75.3 million people being treated in 2021 alone.^3^^,^^6^ PC programs were implemented exclusively in endemic regions after baseline epidemiological surveys. These surveys were conducted to assess the prevalence of infections to evaluate eligibility for PC and to establish the necessary frequency of PC.^5^ For instance, biannual PC was implemented when baseline prevalence exceeded 50%, annual PC was implemented when prevalence ranged from 10% to 49.9%, and biennial PC was implemented when the prevalence fell between 1% and 10%.^5^ However, recent revisions have recognized that schistosomiasis transmission is typically more localized to water contact site catchment areas, and this focality requires more refined smaller spatial implementation scales, such as subdistricts/communities.^7^^,^^8^ The latest WHO guidelines have adjusted the prevalence thresholds for PC to >10%, encompassing individuals across all age groups, including children ages older than 2 years, pregnant women beyond the first trimester, and lactating women.^9^

Following these recommendations, a more precise estimation of the prevalence is needed for better targeting and optimal allocation of resources to the transmission foci. This promises to enhance the efficiency and effectiveness of PC toward the 2030 elimination targets.^7^^,^^8^ WHO has encouraged the conduct of impact assessment studies in implementation units (IUs) after at least 5 years of effective PC (≥75% treatment coverage)^10^ and also, put forth eight distinct methodologies for conducting such assessments, each with its own set of limitations.^10^ Two prominent methodologies, the sentinel and cluster assessment approaches, have traditionally been used for evaluating schistosomiasis endemicity. The sentinel method involves a purposeful selection of fewer representative schools, whereas the cluster method entails a random selection of more schools.^10^ Both approaches assess prevalence at the ecological zone or district level, guiding treatment decisions based on aggregated prevalence (Table 1). Recently, the schistosomiasis practical and precision assessment (SPPA) methodology was developed.^11^ Unlike traditional methods, SPPA evaluates endemicity at the district level but makes treatment decisions at the subdistrict level (Table 1). This strategy aims to streamline the process and save time and resources while aligning with the 10% prevalence threshold outlined in the latest treatment guidelines.^11^

**Table 1:** Overview of the three evaluated impact assessment methodologies

Survey Method	Cluster	Sentinel	Practical
Approach overview	To determine IU-level prevalence and support treatment decisions	To monitor over time prevalence or transmission and progress of intervention in an ecological zone or an area	To identify districts where the same treatment decision is appropriate for all sub-IUs in the district
Methodology	Randomly select schools in each IU, and randomly select children ages 5–14 years old in each school	Purposefully select schools or communities to represent all schools/communities, and randomly select children ages 5–14 years old in each school	Systematically select 15 schools in an IU, ensuring that all sub-IUs are represented, and systematically select children ages 10–14 years old
Evaluation unit	District, group of districts, or ecological zone	Multiple districts or ecological zones	IU
Evaluation metrics	Aggregated prevalence at the evaluation unit	Aggregated prevalence at the multiple districts or ecological zone	Disaggregated prevalence estimate at the sampling sites (i.e., schools)
Decision unit	District, group of districts, ecological zone	Multiple districts or ecological zones	Sub-IU

IU = implementation unit. Districts are also known as local government areas in Nigeria. Subdistricts or sub-IUs are also known as wards in Nigeria.

Given the magnitude and backlog of impact assessment surveys that need to be conducted, programs are looking for the most reliable and sensitive method for adjusting PC decisions considering scarce resources. Therefore, this study evaluated the sensitivity of cluster, practical, and sentinel assessment methodologies for adjusting PC treatment decisions 10 years after implementation in Ondo state, Nigeria.

## MATERIALS AND METHODS

### Study area and settings.

**Figure 1.**
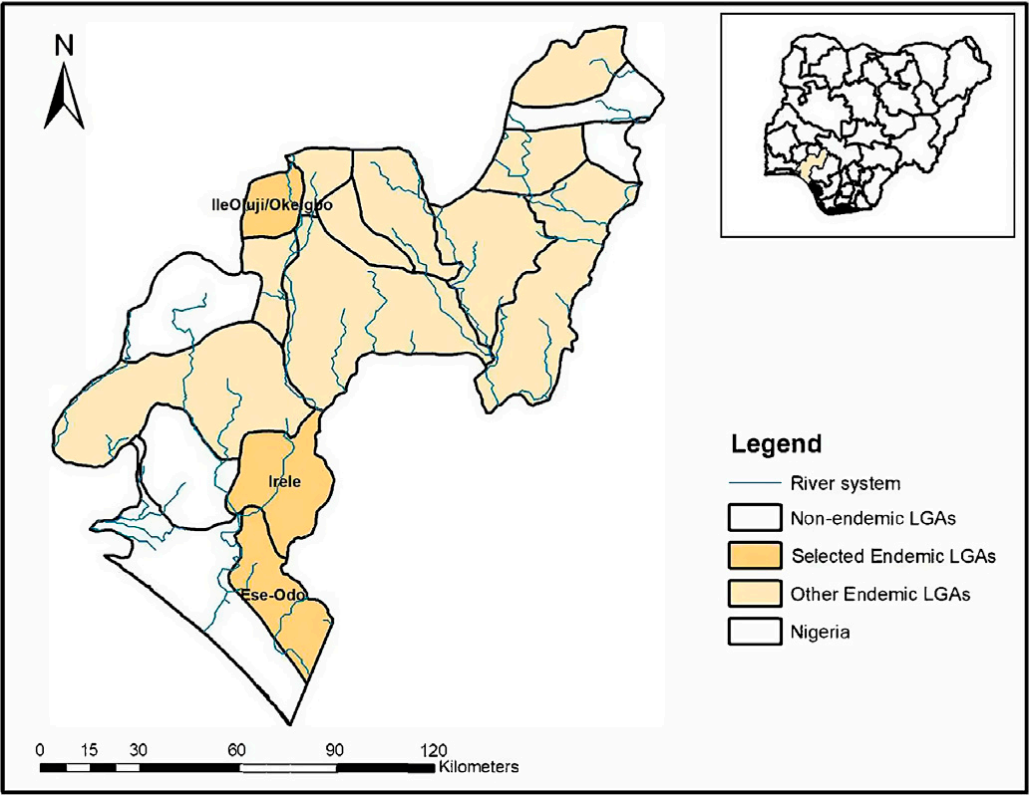
Map of schistosomiasis endemicity in Ondo state: indicating 13 endemic LGAs and selected study locations with Nigeria as an inset. LGA = local government areas.

This study was carried out in the Ese-Odo, Irele, and Ile-Oluiji local government areas (LGAs) of Ondo state, southwestern Nigeria (Figure 1). Ondo state is administratively divided into 18 LGAs, which comprise 206 wards or subdistricts. There are over 3 million inhabitants, with the majority being the Yoruba tribe and Christians. The state is characterized by a tropical climate and three vegetation types (freshwater swamp, rainforest, and Guinea savannah), which in part, contribute to the transmission dynamics of schistosomiasis. Some LGAs bordering the Atlantic Ocean in the south have several ponds, streams, and rivers, which provide a conducive environment for farming and fishing as well as sites for other recreational activities, such as bathing, swimming, and washing clothes.

Baseline epidemiological mapping for schistosomiasis was conducted in 2014, and 13 of the 18 LGAs were found endemic.^12^ The majority of the LGAs (*n* = 9) were categorized as having low endemicity (prevalence between 0.1% and 10%), whereas three LGAs were categorized as having moderate endemicity (prevalence between 10% and 49.9%); only one LGA had a high prevalence (prevalence is greater than 50%).^12^ On average, six effective (>75% coverage) annual rounds of PC have been delivered across the endemic LGAs by the Federal and State Ministry of Health with support from the Ending Neglected Diseases Fund and the Mission to Safe the Helpless, the nongovernmental organization supporting the implementation of PC in the state. All IUs met the minimum number of effective PC rounds required to conduct an impact assessment survey.

### Study design and selection of sampling sites.

Data were collected from March to April 2024 using a cross-sectional sampling design. Questionnaires were administered and samples were collected from school-aged children (SAC) across 45 systematically selected schools (15 per LGA) in three LGAs. Before selecting the sampling sites, LGAs were stratified using a three-step algorithm considering vegetation type, therapeutic coverage in previous PC rounds, and the baseline endemicity category. These criteria form the basis for prioritizing LGAs for assessment purposes. Three LGAs (Ese-Odo, Irele, and Ile-Oluiji) were purposively selected because of their high endemicity at baseline (89% in Ile-Oluiji) and recent epidemiological surveys (54% in Ese-Odo and 84% in Irele).^13^

The sampling sites were government-owned primary schools, and study participants (minors ages 5–14 years old) were chosen in accordance with the WHO guidelines.^5^^,^^11^ All schools within each LGA were listed in a ward or subdistrict. In each ward, one school was randomly selected using paper ballots from every five schools. This approach ensures that each subdistrict has an unbiased and equal chance of selection while also providing adequate geographic representation across the district and its subdistricts.^11^

### Sample size determination and selection of study participants.

WHO recommends the recruitment of 50 SAC between the ages of 5 and 14 years old while assessing worm burden and the impact of PC.^5^ However, recent guidelines for utilizing the SPPA tool recommend recruiting 30 late SAC ages 10–14 years old.^11^ Hence, we adopted both recommendations, aiming for at least 50 SAC per school. This comprised 30 late SAC between ages 10–14 years old and 20 early SAC ages 5–9 years old. Thus, the minimum sample size targeted was 2,250: that is, 750 SAC per LGA. However, recruitment extended beyond this estimate to accommodate for refusal.

### Selection of study participants and exclusion criteria.

Participants were recruited from their school premises with the assistance of teachers. Children ages 5–9 years old were selected from the first, second, and third primary school classes, with six to seven children of both sexes (male and female) recruited per class. Children ages 10–14 years old were recruited from upper classes corresponding to the fourth, fifth, and sixth primary school years. A minimum of 10 children (five males and five females) were recruited to fulfil the sampling targets in each of the upper classes. The selection was carried out systematically, with the children forming a line based on their class and gender. Each child was assigned a chronological number, and the sampling interval was calculated by dividing the number of children in the line by the required number of boys or girls to be sampled per class. The first child was chosen through paper balloting, and the subsequent selection was determined by adding the sampling interval to the position of the previous child. This process continued until the target was reached. Where the target sample size for a class was not achieved, efforts were made to recruit additional children from the same class, school, or community. The exclusion criteria were children outside the target age range (5–14 years old), those who were unwell, and those whose parents declined to participate.

### Questionnaire administration.

This study used a closed-ended electronic questionnaire to gather epidemiological data from the consenting participants. Three questionnaires, 1) a school field form, 2) an individual questionnaire, and 3) a snail survey field form, were designed in electronic form and administered with Android or iOS phones. Before this survey, all questionnaires had been validated and used for over a decade by our broader team in similar epidemiological surveys.^14^^–^^16^ The school field collected information on school names; coordinates; enrolment figures; and the availability of water, sanitation, and hygiene (WASH) facilities. Conversely, the individual form documented demographic data, including age, sex, and parental occupation, as well as information on WASH facilities at the household level, water contact practices, participation in PC programs, fidelity of PC implementation, and parasitological data from the examined samples. Copies of the questionnaire are available at https://zenodo.org/records/11080490. All of the data were collected electronically and promptly transferred to a remote backup server after each interview. Interviews were conducted in the Yoruba language and held confidentially in a private setting unless the interviewee required the presence of a legal guardian or parent.

### Collection of stool and urine samples.

Before administering the questionnaire, the enrolled participants who had completed the informed consent forms were given unique identification tags. These tags were affixed to consent forms, sterile specimen bottles, microscopic slides, and result sheets to ensure quality control. Each participant received two sterile specimen bottles for urine and stool samples on the day of collection along with an applicator stick, a plain sheet of paper, tissue paper, and soap to aid in the process of voiding feces and cleaning up afterward. The participants were instructed to provide fecal, midstream, and terminal urine samples before noon. The processes of voiding urine and stool collection were supervised by a teacher and a field team representative. Samples were collected within the first hour of the specimen bottle distribution. Bar soaps were provided as incentives to encourage positive hygiene practices in a noncoercive manner, ensuring that they did not influence the participants’ decision to participate or decline participation in the research.

### Parasitological assessment of stool and urine samples.

The samples were processed on-site within 2 hours of collection using a mobile laboratory within each school. The urine filtration method was used to recover *S. hematobium* eggs from the urine samples.^17^ Briefly, 10 mL of the urine sample was vigorously shaken before being passed through a polycarbonate membrane filter with a 20-µm mesh size. Subsequently, the filter was placed on a clean microscope slide and examined under a microscope using ×10 and ×40 objective lenses to identify eggs with characteristic terminal spines.^17^ Stool specimens were processed using the Kato–Katz technique. A single thick smear was prepared from each stool sample and allowed to clear for 30 minutes before microscopic examination of *S. mansoni*.^17^ Each slide was carefully re-examined, and egg counts were verified by another microscopist to ensure quality assurance. The intensity of infection (eggs per gram) from the Kato–Katz smears was multiplied by a factor of 24 before being recorded on the participants’ sheets. Positive participants were defined as those with at least one egg count per slide for any parasite under consideration.

## STATISTICAL ANALYSES

The data collection process was remotely monitored using the KoboCollect platform. Field supervisors also recorded summarized daily entries in a closed-tracking tool, facilitating the triangulation of electronic uploads and enabling prompt resolution of discrepancies. Three distinct tracking tools were used throughout the study: one for each of the school field forms and parasitology and malacology components. All variables were imported into R Studio v. 4.3.2 (R Foundation, Vienna, Austria) for the analysis. Initially, these variables were transformed, categorical responses were converted into factors, and numeric responses were converted into integers. Subsequently, responses were encoded into binary codes using the corresponding questionnaires and data codes.

For the prevalence data, entries were initially treated as numeric values and used to estimate the overall infection status by using the “rowSums” function in R. For instance, the overall schistosomiasis infection status was determined by summing all of the positive responses for *S. mansoni* and *S. hematobium*. Participants with counts greater than or equal to one were recorded as positive for overall schistosomiasis infection. Regarding infection intensity, participants with *S. hematobium* egg counts between 1 and 49 were classified as having a light infection, whereas those with counts ≥50 were considered to have a heavy infection.^12^ Conversely, for *S. mansoni*, infections were categorized as light with egg counts between 1 and 99, moderate with counts between 100 and 399, and heavy with counts ≥400 or more.^12^

For the statistical analysis, we adhered to a systematic approach. Initially, descriptive statistics were used to summarize all of the dependent and response variables as proportions. Subsequently, associations were investigated using χ^2^ statistics, primarily focusing on demographic data, such as age and sex. Following this initial analysis, we conducted three independent analyses based on three assessment methodologies: cluster, sentinel, and practical. The cluster methodology analysis involved all 15 schools and participants ages 5–14 years old. However, for practical assessment, we filtered only participants ages 10–14 years old. In contrast, the sentinel assessment used a stratification approach for all 15 schools within each LGA, considering factors such as the ecological zone, endemicity profile, and therapeutic coverage. This stratification facilitated the random selection of 5 representative schools from the sampled 15, using the “unique” and “sample” base functions in R. Subsequently, the dataset was filtered to include only the selected schools, and the analysis was conducted for all participants ages 5–14 years old.

Finally, we calculated the percentage change in prevalence across the three assessment methodologies to illustrate how the data reduction affected the observed prevalence. The prevalence for each approach was estimated and compared with that of the cluster-based approach as a reference because the cluster method was used in the initial baseline survey. All data analyses were conducted separately for each unit. Associations between variables were assessed at a 95% significance level, and where applicable, Clopper–Pearson CIs were computed for prevalence estimates. Additional files containing the dataset and analysis code are attached at https://zenodo.org/records/11080490.

## RESULTS

### Sex and age profile of study participants.

Table 2 shows the sex and age profile of study participants. In total, 2,093 SAC from 15 schools in each of the three LGAs participated in this study: 722 from Ese-Odo, 750 from Irele, and 621 from Ile-Oluiji. The recruitment exceeded 80% of the estimated target size for each LGA. The gender distribution of participants did not significantly differ across the LGAs; however, there were slightly more male than female participants in Ese-Odo (53.6% versus 46.4%, *P* = 0.90), Irele (50.1% versus 49.9%, *P* = 0.67), and Ile-Oluiji (51.4% versus 48.6%, *P* = 0.48). Regarding age, most participants fell within the 10–14 age categories compared with those younger than 10 years of age. Significant variations in the participant distribution by age were observed for Ese-Odo (54.6% versus 45.4%, *P* = 0.014) and Ile-Oluiji (52.7% versus 47.3%, *P* = 0.001).

**Table 2:** Sex and age characteristics of study participants

School Identification	Local Government Areas														
	Ese-Odo					Irele					Ile-Oluiji				
	*N*	Sex, *n* (%)		Age in Years, *n* (%)		*N*	Sex, *n* (%)		Age in Years, *n* (%)		*N*	Sex, *n* (%)		Age in Years, *n* (%)	
		Female	Male	5–9	10–14		Female	Male	5–9	10–14		Female	Male	5–9	10–14
1	50	25 (50.0)	25 (50.0)	22 (44.0)	28 (56.0)	49	22 (44.9)	27 (55.1)	18 (36.7)	31 (63.3)	24	10 (41.7)	14 (58.3)	19 (79.2)	5 (20.8)
2	49	20 (40.8)	29 (59.2)	18 (36.7)	31 (63.3)	48	22 (45.8)	26 (54.2)	20 (41.7)	28 (58.3)	35	14 (40.0)	21 (60.0)	17 (48.6)	18 (51.4)
3	48	23 (47.9)	25 (52.1)	20 (41.7)	28 (58.3)	50	32 (64.0)	18 (36.0)	20 (40.0)	30 (60.0)	31	22 (71.0)	9 (29.0)	15 (48.4)	16 (51.6)
4	44	17 (38.6)	27 (61.4)	22 (50.0)	22 (50.0)	51	25 (49.0)	26 (51.0)	22 (43.1)	29 (56.9)	50	22 (44.0)	28 (56.0)	18 (36.0)	32 (64.0)
5	42	21 (50.0)	21 (50.0)	12 (28.6)	30 (71.4)	50	25 (50.0)	25 (50.0)	28 (56.0)	22 (44.0)	44	23 (52.3)	21 (47.7)	16 (36.4)	28 (63.6)
6	50	22 (44.0)	28 (56.0)	20 (40.0)	30 (60.0)	50	32 (64.0)	18 (36.0)	17 (34.0)	33 (66.0)	21	7 (33.3)	14 (66.7)	4 (19.0)	17 (81.0)
7	50	26 (52.0)	24 (48.0)	20 (40.0)	30 (60.0)	59	29 (49.2)	30 (50.8)	26 (44.1)	33 (55.9)	51	25 (49.0)	26 (51.0)	28 (54.9)	23 (45.1)
8	50	27 (54.0)	23 (46.0)	31 (62.0)	19 (38.0)	51	25 (49.0)	26 (51.0)	21 (41.2)	30 (58.8)	50	28 (56.0)	22 (44.0)	29 (58.0)	21 (42.0)
9	50	23 (46.0)	27 (54.0)	22 (44.0)	28 (56.0)	49	24 (49.0)	25 (51.0)	20 (40.8)	29 (59.2)	37	19 (51.4)	18 (48.6)	18 (48.6)	19 (51.4)
10	51	25 (49.0)	26 (51.0)	24 (47.1)	27 (52.9)	48	24 (50.0)	24 (50.0)	18 (37.5)	30 (62.5)	43	18 (41.9)	25 (58.1)	22 (51.2)	21 (48.8)
11	49	23 (46.9)	26 (53.1)	26 (53.1)	23 (46.9)	50	25 (50.0)	25 (50.0)	20 (40.0)	30 (60.0)	50	24 (48.0)	26 (52.0)	17 (34.0)	33 (66.0)
12	50	25 (50.0)	25 (50.0)	25 (50.0)	25 (50.0)	50	25 (50.0)	25 (50.0)	22 (44.0)	28 (56.0)	37	17 (45.9)	20 (54.1)	21 (56.8)	16 (43.2)
13	48	18 (37.5)	30 (62.5)	15 (31.0)	33 (68.8)	51	22 (43.1)	29 (56.9)	21 (41.2)	29 (58.0)	49	22 (44.9)	27 (55.1)	19 (38.6)	30 (61.2)
14	43	16 (37.2)	27 (62.8)	19 (44.2)	24 (55.8)	50	24 (48.0)	26 (52.0)	21 (42.0)	29 (58.0)	50	24 (48.0)	26 (52.0)	26 (52.0)	24 (48.0)
15	48	24 (50.0)	24 (50.0)	32 (66.7)	16 (33.3)	44	18 (40.9)	26 (59.1)	14 (31.8)	30 (68.2)	49	27 (55.1)	22 (44.9)	25 (51.0)	24 (49.0)
Total	722	335 (46.4)	387 (53.6)	328 (45.4)	394 (54.6)	750	374 (49.9)	376 (50.1)	308 (41.1)	442 (58.9)	621	302 (48.6)	319 (51.4)	294 (47.3)	327 (52.7)
*P*-value	–	0.90	–	0.014	–	–	0.67	–	0.87	–	–	0.48	–	0.001	–

### Prevalence and intensity of schistosomiasis among study participants by LGAs.

The prevalence of schistosomiasis in the study population is presented in Table 3. Approximately 93% of the participants enrolled in this study returned urine or stool samples for examination. In Ese-Odo, the overall prevalence of schistosomiasis decreased from 1.3% (95% CI: 0.5–3.3) at baseline to 0.1% (95% CI: 0.01–0.95; d = −92.3%, *P* = 0.03), and in Ile-Oluiji, the prevalence decreased from 58.0% (95% CI: 53.9–62.1) at baseline to 1.8% (95% CI: 0.9–3.3; d = −97%, *P* = 0.00). However, in Irele, the prevalence increased from 3.0% (95% CI: 1.6–5.6) to 5.3% (95% CI: 3.8–7.3; d = 66%, *P* = 0.13) (Table 3). In Ese-Odo, the proportion of participants with heavy egg intensity for *S. hematobium* was 0.1%, whereas none of the participants had heavy egg intensity for *S. mansoni*. In Irele, 2.3% of the participants had heavy egg intensity for* S. hematobium*, and 0.2% had heavy egg intensity for *S. mansoni*. Similarly, in Ile-Oluiji, the proportion of participants with heavy egg intensity for *S. hematobium* was 0.1%, and none of the participants had heavy egg intensity for *S. mansoni* (Table 3).

**Table 3:** Prevalence and intensity of schistosomiasis among study participants by local government areas

Survey Type	Ese-Odo		Irele		Ile-Oluiji	
	Baseline	Impact	Baseline	Impact	Baseline	Impact
Year	2011	2024	2011	2024	2011	2024
Methodology	Sentinel	Cluster	Sentinel	Cluster	Sentinel	Cluster
Number of schools	6	15	6	15	5	15
Age group, years	5–14	5–14	5–14	5–14	5–14	5–14
Number examined*	305	683	301	663	546	608
Number infected*	4	1	9	35	317	11
Overall prevalence (95% CI)*	1.3% (0.5–3.3)	0.1% (0.01–0.95)	3.0% (1.6–5.6)	5.3% (3.8–7.3)	58.0% (53.9–62.1)	1.8% (0.95–3.3)
Proportion with heavy intensity for *Schistosoma haematobium*^†^	ND	1/712 (0.1%)	ND	17/736 (2.3%)	ND	1/615 (0.1%)
Proportion with heavy intensity for *Schistosoma mansoni*^‡^	ND	0/691 (0.0%)	ND	1/668 (0.15%)	ND	0/613 (0.0%)

ND = no data.

* Estimation was based on the total number of participants who provided both urine and stool samples.

^†^ Estimation was based on the number of participants who provided urine samples.

^‡^ Estimation was based on the number of participants who provided stool samples.

### Prevalence of schistosomiasis based on cluster, practical, and sentinel assessment methodologies.

Tables 4–6 show the endemicity of schistosomiasis in LGAs based on the three assessment methodologies. In Ese-Odo, the cluster methodology indicated an aggregated schistosomiasis prevalence of 0.1%, with one school reporting a site prevalence of 2.0%. The practical methodology showed an increased aggregated prevalence of 0.3% with a site prevalence of 3.6%. However, no prevalence was found across five randomly selected schools using the sentinel methodology. All of the infections in Ese-Odo were attributed to *S. hematobium* (Table 4). In Irele LGA, the cluster methodology revealed an aggregated prevalence of 5.3% for overall schistosomiasis across 6 of 15 schools, with a species prevalence of 5.0% across 5 schools and 0.3% across 2 schools for *S. hematobium* and *S. mansoni*, respectively. The practical methodology showed an aggregated prevalence of 5.8%, with a species prevalence of 5.3% across five schools and 0.3% in one school for *S. hematobium* and *S. mansoni*, respectively. Sentinel methodology also indicated an aggregated prevalence of 5.4% across the two schools, with a species prevalence of 6.5% across the two schools for *S. hematobium* only (Table 5). In Ile-Oluiji LGA, the cluster methodology showed an aggregated prevalence of 1.8% for overall schistosomiasis across 5 of 15 schools, with a species prevalence of 1.8% across 5 schools for *S. hematobium* only. The practical methodology revealed an aggregated prevalence of 2.2%, with a species prevalence of 2.2% across the four schools for *S. hematobium* only. Sentinel methodology also showed an aggregated prevalence of 1.5% across the two schools, with a species prevalence of 6.5% across the two schools for *S. hematobium* only (Table 6).

**Table 4:** Prevalence of schistosomiasis based on cluster, practical, and sentinel assessment methodologies in the Ese-Odo local government area

School Identification	Impact Assessment Methodologies																	
	Cluster*						Practical^†^						Sentinel^‡^					
	Urine		Stool		Both Samples		Urine		Stool		Both Samples		Urine		Stool		Both Samples	
	*N*	Sh	*N*	Sm	*N*	Sh + Sm	*N*	Sh	*N*	Sm	*N*	Sh + Sm	*N*	Sh	*N*	Sm	*N*	Sh + Sm
		*n* (%)		*n* (%)		*n* (%)		*n* (%)		*n* (%)		*n* (%)		*n* (%)		*n* (%)		*n* (%)
1	49	1 (2.0)	50	0 (0)	49	1 (2.0)	28	1 (3.6)	28	0 (0)	28	1 (3.6)	–	–	–	–	–	–
2	49	0 (0)	42	0 (0)	42	0 (0)	31	0 (0)	28	0 (0)	28	0 (0)	49	0 (0)	42	0 (0)	42	0 (0)
3	48	0 (0)	42	0 (0)	41	0 (0)	26	0 (0)	24	0 (0)	23	0 (0)	–	–	–	–	–	–
4	44	0 (0)	42	0 (0)	42	0 (0)	22	0 (0)	20	0 (0)	20	0 (0)	–	–	–	–	–	–
5	42	0 (0)	42	0 (0)	42	0 (0)	30	0 (0)	30	0 (0)	30	0 (0)	–	–	–	–	–	–
6	50	0 (0)	50	0 (0)	50	0 (0)	30	0 (0)	30	0 (0)	30	0 (0)	50	0 (0)	50	0 (0)	50	0 (0)
7	50	0 (0)	50	0 (0)	50	0 (0)	30	0 (0)	30	0 (0)	30	0 (0)	–	–	–	–	–	–
8	50	0 (0)	50	0 (0)	50	0 (0)	19	0 (0)	19	0 (0)	19	0 (0)	–	–	–	–	–	–
9	50	0 (0)	50	0 (0)	50	0 (0)	28	0 (0)	28	0 (0)	28	0 (0)	50	0 (0)	50	0 (0)	50	0 (0)
10	49	0 (0)	50	0 (0)	49	0 (0)	26	0 (0)	27	0 (0)	26	0 (0)	49	0 (0)	50	0 (0)	49	0 (0)
11	48	0 (0)	38	0 (0)	37	0 (0)	23	0 (0)	20	0 (0)	20	0 (0)	–	–	–	–	–	–
12	48	0 (0)	48	0 (0)	46	0 (0)	24	0 (0)	23	0 (0)	22	0 (0)	–	–	–	–	–	–
13	48	0 (0)	48	0 (0)	48	0 (0)	33	0 (0)	33	0 (0)	33	0 (0)	48	0 (0)	48	0 (0)	48	0 (0)
14	40	0 (0)	41	0 (0)	38	0 (0)	21	0 (0)	23	0 (0)	20	0 (0)	–	–	–	–	–	–
15	48	0 (0)	48	0 (0)	48	0 (0)	16	0 (0)	16	0 (0)	16	0 (0)	–	–	–	–	–	–
Total	711	1 (0.1)	691	0 (0)	683	1 (0.1)	387	1 (0.3)	379	0 (0)	373	1 (0.3)	246	0 (0)	240	0 (0)	239	0 (0)
*P*-value	–	–	–	–	–	–	–	0.54	–	–	–	0.58	–	–	–	–	–	–

*N* = number examined; *n* = number of positives; Sh = *Schistosoma hematobium*; Sm = *Schistosoma mansoni*.

* Children ages 5–14 years old examined across 15 schools.

^†^ Children ages 10–14 years old examined across 15 schools.

^‡^ Children ages 5–14 years old examined across five schools.

**Table 5:** Prevalence of schistosomiasis based on cluster, practical and sentinel assessment methodologies in the Irele local government area

School Identification	Impact Assessment Methodologies																	
	Cluster*						Practical^†^						Sentinel^‡^					
	Urine		Stool		Both Samples		Urine		Stool		Both Samples		Urine		Stool		Both Samples	
	*N*	Sh	*N*	Sm	*N*	Sh + Sm	*N*	Sh	*N*	Sm	*N*	Sh + Sm	*N*	Sh	*N*	Sm	*N*	Sh + Sm
		*n* (%)		*n* (%)		*n* (%)		*n* (%)		*n* (%)		*n* (%)		*n* (%)		*n* (%)		*n* (%)
1	49	0 (0)	49	0 (0)	49	0 (0)	31	0 (0)	31	0 (0)	31	0 (0)	49	0 (0)	49	0 (0)	49	0 (0)
2	48	0 (0)	47	1 (2.1)	47	1 (2.1)	28	0 (0)	28	1 (3.6)	28	1 (3.6)	–	–	–	–	–	–
3	50	0 (0)	48	0 (0)	48	0 (0)	30	0 (0)	29	0 (0)	29	0 (0)	–	–	–	–	–	–
4	50	0 (0)	50	0 (0)	50	0 (0)	28	0 (0)	28	0 (0)	28	0 (0)	–	–	–	–	–	–
5	50	0 (0)	50	0 (0)	50	0 (0)	22	0 (0)	22	0 (0)	22	0 (0)	50	0 (0)	50	0 (0)	50	0 (0)
6	50	10 (20.0)	50	0 (0)	50	10 (20.0)	33	7 (21.2)	33	0 (0)	33	7 (21.2)	–	–	–	–	–	–
7	54	0 (0)	41	0 (0)	40	0 (0)	32	0 (0)	27	0 (0)	27	0 (0)	–	–	–	–	–	–
8	51	4 (7.8)	51	1 (2.0)	51	4 (7.8)	30	2 (6.7)	30	0 (0)	30	2 (6.7)	–	–	–	–	–	–
9	49	1 (2.0)	49	0 (0)	49	1 (2.0)	29	1 (3.4)	29	0 (0)	29	1 (3.4)	49	1 (2.0)	49	0 (0)	49	1 (2.0)
10	48	0 (0)	48	0 (0)	48	0 (0)	30	0 (0)	30	0 (0)	30	0 (0)	–	–	–	–	–	–
11	46	0 (0)	0	0 (0)	0	0 (0)	27	0 (0)	0	0 (0)	0	0 (0)	–	–	–	–	–	–
12	50	0 (0)	50	0 (0)	50	0 (0)	28	0 (0)	28	0 (0)	28	0 (0)	–	–	–	–	–	–
13	49	15 (30.6)	46	0 (0)	44	12 (27.3)	28	7 (25.0)	28	0 (0)	26	6 (23.1)	49	15 (30.6)	46	0 (0)	44	12 (27.3)
14	50	0 (0)	48	0 (0)	48	0 (0)	29	0	27	0 (0)	27	0 (0)	50	0 (0)	48	0 (0)	48	0 (0)
15	42	7 (16.7)	41	0 (0)	39	7 (17.9)	28	6 (21.4)	28	0 (0)	26	6 (23.1)	–	–	–	–	–	–
Total	736	37 (5.0)	668	2 (0.3)	663	35 (5.3)	423	23 (5.3)	398	1 (0.3)	394	23 (5.8)	247	16 (6.5)	242	0 (0)	240	13 (5.4)
*P*-value	–	0.001	–	0.55	–	0.001	–	0.001	–	0.43	–	0.001	–	0.001	–	–	–	0.001

*N* = number examined; *n* = number of positives; Sh = *Schistosoma hematobium*; Sm = *Schistosoma mansoni*.

* Children ages 5–14 years old examined across 15 schools.

^†^ Children ages 10–14 years old examined across 15 schools.

^‡^ Children ages 5–14 years old examined across five schools.

**Table 6:** Prevalence of schistosomiasis based on cluster, practical, and sentinel assessment methodologies in the Ile-Oluiji local government area

School Identification	Impact Assessment Methodologies																	
	Cluster*						Practical^†^						Sentinel^‡^					
	Urine		Stool		Both Samples		Urine		Stool		Both Samples		Urine		Stool		Both Samples	
	*N*	Sh	*N*	Sm	*N*	Sh + Sm	*N*	Sh	*N*	Sm	*N*	Sh + Sm	*N*	Sh	*N*	Sm	*N*	Sh + Sm
		*n* (%)		*n* (%)		*n* (%)		*n* (%)		*n* (%)		*n* (%)		*n* (%)		*n* (%)		*n* (%)
1	24	0 (0)	24	0 (0)	24	0 (0)	5	0 (0)	5	0 (0)	5	0 (0)	–	–	–	–	–	–
2	35	1 (2.9)	32	0 (0)	31	1 (3.2)	18	0 (0)	15	0 (0)	15	0 (0)	35	1 (2.9)	32	0 (0)	31	1 (3.2)
3	31	0 (0)	30	0 (0)	30	0 (0)	16	0 (0)	16	0 (0)	16	0 (0)	31	0 (0)	30	0 (0)	30	0 (0)
4	49	0 (0)	49	0 (0)	49	0 (0)	31	0 (0)	31	0 (0)	31	0 (0)	–	–	–	–	–	–
5	43	0 (0)	44	0 (0)	43	0 (0)	27	0 (0)	28	0 (0)	27	0 (0)	43	0 (0)	44	0 (0)	43	0 (0)
6	21	1 (4.8)	21	0 (0)	20	1 (4.8)	17	1 (5.9)	17	0 (0)	17	1 (5.9)	–	–	–	–	–	–
7	41	5 (9.8)	51	0 (0)	46	5 (9.8)	23	2 (13.0)	23	0 (0)	23	3 (13.0)	–	–	–	–	–	–
8	49	0 (0)	50	0 (0)	49	0 (0)	21	0 (0)	21	0 (0)	21	0 (0)	–	–	–	–	–	–
9	37	0 (0)	36	0 (0)	36	0 (0)	19	0 (00	18	0 (0)	18	0 (0)	–	–	–	–	–	–
10	41	2 (4.9)	42	0 (0)	38	2 (5.0)	21	1 (4.8)	20	0 (0)	20	1 (5.0)	41	2 (4.9)	42	0 (0)	38	2 (5.0)
11	50	2 (4.0)	50	0 (0)	48	2 (4.0)	33	2 (6.1)	33	0 (0)	33	2 (6.1)	–	–	–	–	–	–
12	37	0 (0)	37	0 (0)	37	0 (0)	16	0 (0)	16	0 (0)	16	0 (0)	–	–	–	–	–	–
13	48	0 (0)	49	0 (0)	48	0 (0)	29	0 (0)	30	0 (0)	29	0 (0)	–	–	–	–	–	–
14	50	0 (0)	49	0 (0)	49	0 (0)	24	0 (0)	23	0 (0)	23	0 (0)	–	–	–	–	–	–
15	49	0 (0)	49	0 (0)	49	0 (0)	24	0 (0)	24	0 (0)	24	0 (0)	50	0 (0)	49	0 (0)	49	0 (0)
Total	615	11 (1.8)	613	0 (0)	608	11 (1.8)	319	7 (2.2)	320	0 (0)	318	7 (2.2)	200	3 (1.5)	197	0 (0)	194	3 (1.5)
*P*-value	–	0.005	–	–	–	0.006		0.076	–	–	–	0.085		0.24	–	–	–	0.23

*N* = number examined; *n* = number of positives; Sh = *Schistosoma hematobium*; Sm = *Schistosoma mansoni*.

* Children ages 5–14 years old examined across 15 schools.

^†^ Children ages 10–14 years old examined across 15 schools.

^‡^ Children ages 5–14 years old examined across five schools.

### Percentage change in prevalence across the three sampling approaches in each of the study LGAs.

Table 7 illustrates the differences in prevalence across the three assessment methodologies within each study’s LGAs. The prevalences based on the cluster, practical, and sentinel methodologies were as follows: 0.1%, 0.3%, and 0.0% in Ese Odo; 5.3%, 5.8%, and 5.4% in Irele; and 1.8%, 2.2%, and 1.5% in Ile-Oluiji, respectively. Using the cluster approach as a reference point, the sentinel approach exhibited a –100% decrease in prevalence percentage (*P* = 0.554) compared with the practical assessment, which showed an increase in percentage change of 83.1% (*P* = 0.664) in Ese-Odo. Similarly, in Irele, the sentinel approach demonstrated a 2.6% increase in prevalence percentage (*P* = 0.938) compared with the practical assessment, which showed an increase in percentage change of 10.6% (*P* = 0.715). In Ile-Oluiji, the sentinel approach displayed a –14.5% decrease in the prevalence percentage (*P* = 0.874) relative to the practical assessment, which showed an increase in the percentage change of 21.7% (*P* = 0.687).

**Table 7:** Percentage change in prevalence across the three sampling approaches in each of the study local government areas

Methodology	Ese-Odo					Irele					Ile-Oluiji				
	NE	NI	%	d	*P*-Value*	NE	NI	%	d	*P-*Value*	NE	NI	%	d	*P-*Value*
Cluster	683	1	0.1	–	–	663	35	5.28	–	–	608	11	1.81	–	–
Practical	373	1	0.3	83.1	0.664	394	23	5.83	10.6	0.715	318	7	2.20	21.7	0.687
Sentinel	239	0	0.0	−100	0.554	240	13	5.42	2.61	0.938	194	3	1.54	−14.5	0.874

%= prevalence percentage; d = percentage change in prevalence with the cluster method as reference; NE = number examined; NI = number infected. Cluster is considered the baseline because it has a larger sample size.

* The χ^2^ tests are between cluster (reference) and other methods.

### Programmatic interpretation of prevalence data based on the three assessment methodologies.

**Figure 2.**
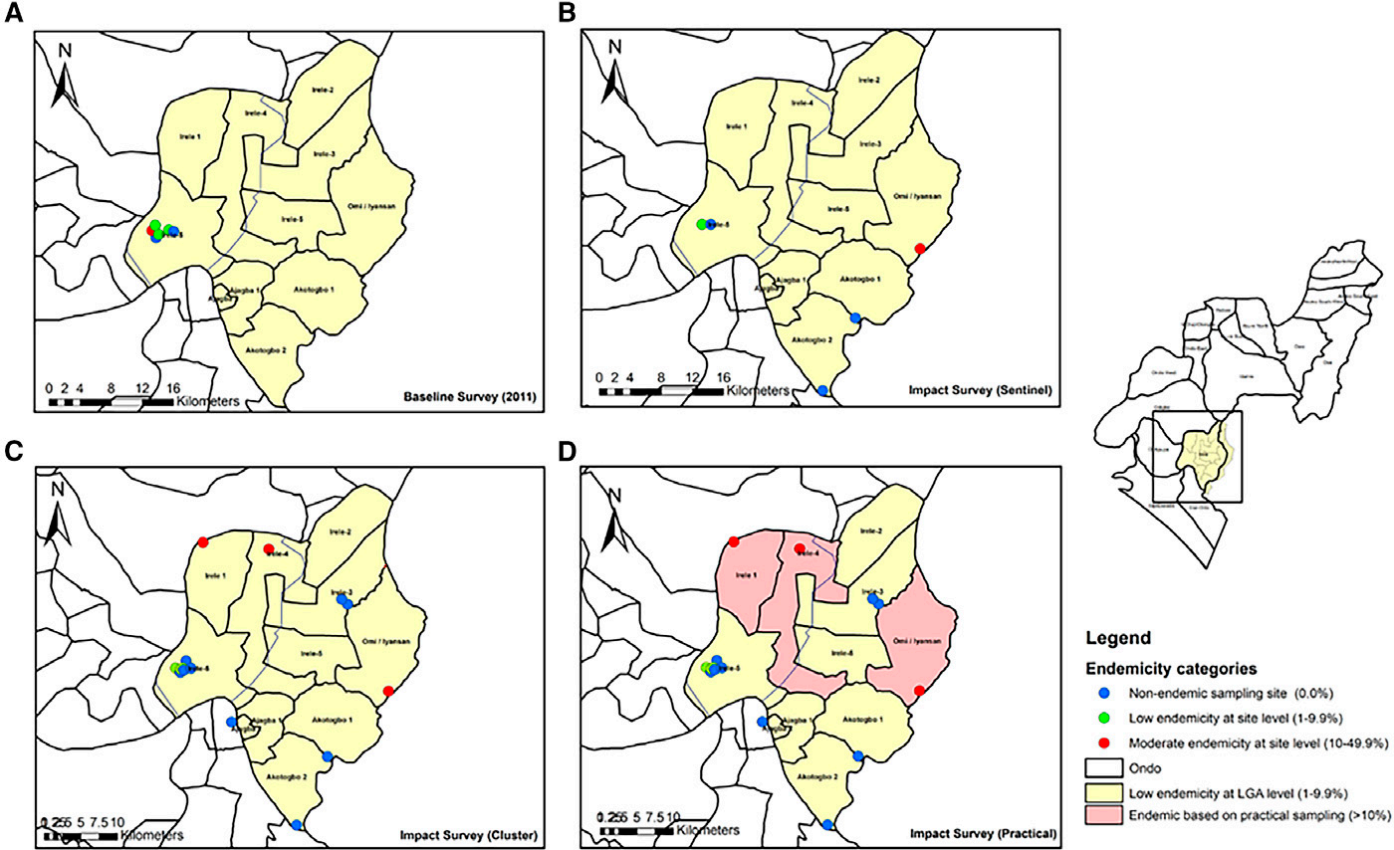
Comparative impact assessment of schistosomiasis endemicity maps in Irele LGA: evaluating the sentinel, cluster, and practical survey methods against baseline data. LGA = local government areas.

**Figure 3.**
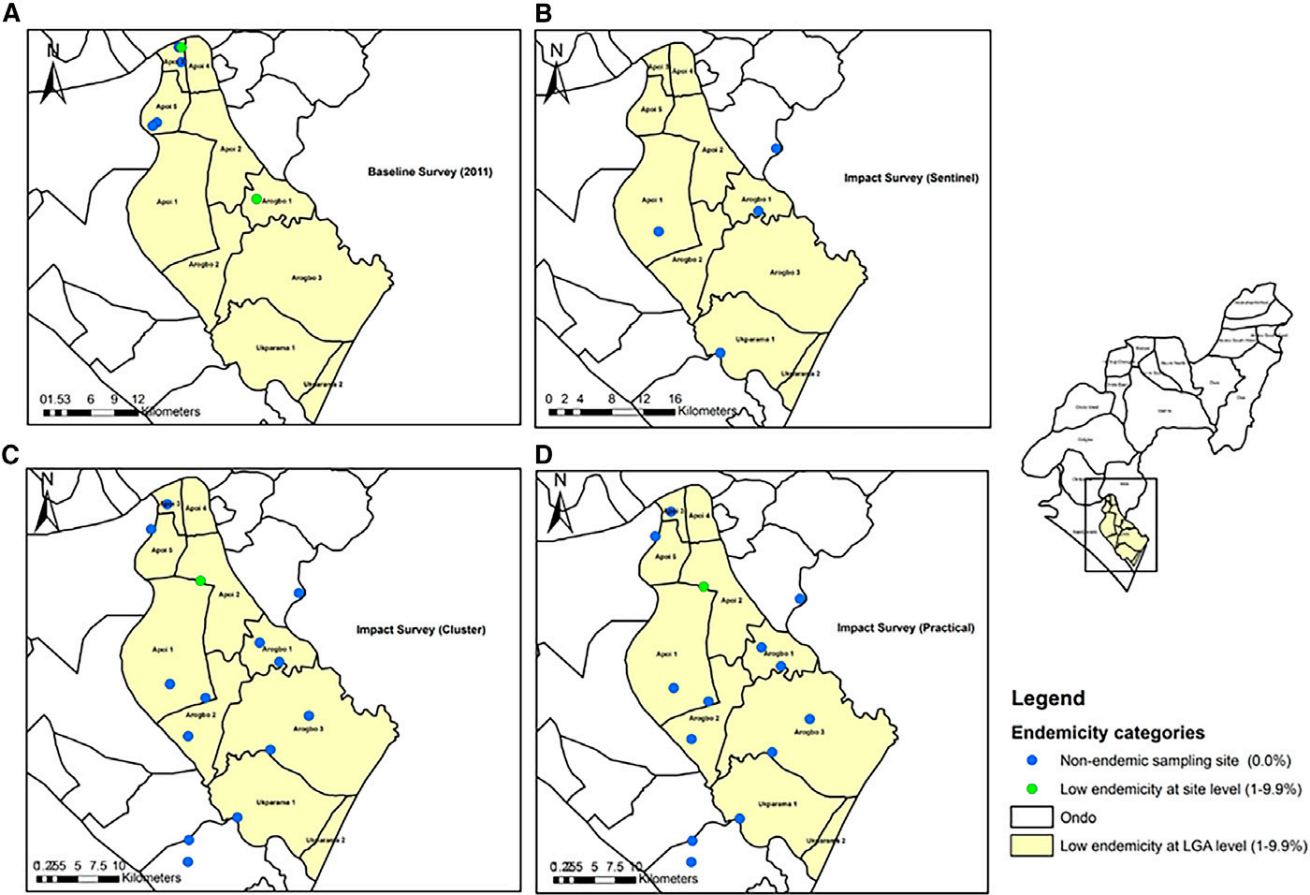
Comparative impact assessment of schistosomiasis endemicity maps in Ese-Odo LGA: evaluating the sentinel, cluster, and practical survey methods against baseline data. LGA = local government areas.

**Figure 4.**
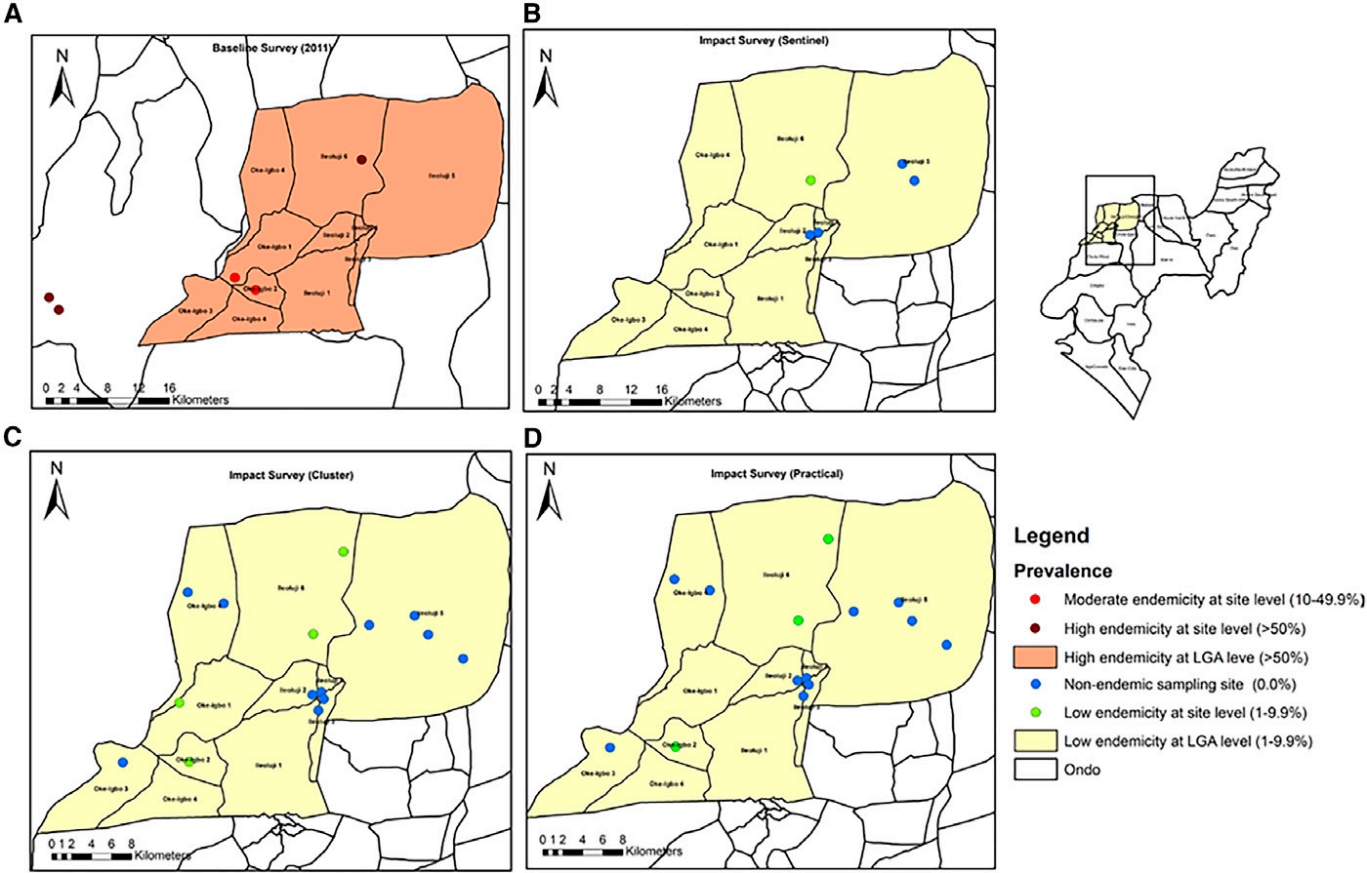
Comparative impact assessment of schistosomiasis endemicity maps in Ile-Oluiji LGA: evaluating the sentinel, cluster, and practical survey methods against baseline data. LGA = local government areas.

Figures 2–4 present the endemicity maps of the study LGAs. In 2011, the Irele LGA was categorized as having a low endemicity status, with an aggregated prevalence below 10%, and only one of the six sampled schools exhibited a prevalence of 10% (Figure 2A). However, the current study (Figure 2B) also revealed an aggregate prevalence below 10% based on cluster sampling (Figure 2B), with three schools reporting a site prevalence >10%. Similarly, findings from the sentinel approach indicated an aggregated prevalence of <10%, with only one of five schools reporting a prevalence of >10% (Figure 2C). The implication of these findings as indicated by the guidelines for the sentinel and cluster approaches suggests that Irele does not require PC because the aggregated prevalence is below 10%. However, with the practical approach, 3 of the 15 sampling sites had prevalence rates above 10%, implying heterogeneous transmission in the LGA with hot spots in Irele 3, Irele 4, and Omi Iyasan and hence, confirming that the LGA requires an annual PC (Figure 2D).

Figure 3 shows the endemicity map of Ese-Odo LGA. In 2011, LGA was categorized as having a low endemicity status, with an aggregated prevalence below 10%, and only two of the five sampled schools exhibited a prevalence between 1% and 9.9% (Figure 3A). However, the current study (Figure 3B) also revealed an aggregate prevalence below 10% based on cluster and sentinel sampling (Figure 3B and C), with only one school reporting a site prevalence between 1% and 9.9%. The implication of these findings as indicated by the guidelines for the sentinel and cluster approaches suggests that Ese-Odo does not require PC because the aggregated prevalence is below 10%. Also, with the practical approach (Figure 3D), none of the 15 sampling sites had prevalence rates above 10%, implying a homogeneously low prevalence of the LGA and confirming that the LGA does not require PC (Figure 3D).

Figure 4 presents an endemicity map of Ile-Oluiji LGA. In 2011, Ile-Oluiji was categorized as highly endemic with an aggregated prevalence of >50% (Figure 4A). However, the current study (Figure 4B) revealed an aggregate prevalence below 10% based on cluster and sentinel sampling (Figure 4B and C), with only one and four schools reporting site prevalence between 1% and 9.9% for sentinel and cluster surveys, respectively. The implication of these findings as indicated by the guidelines for the sentinel and cluster approaches suggests that Ile-Oluiji does not require PC because the aggregated prevalence is below 10%. Also, with the practical approach (Figure 4D), none of the 15 sampling sites had prevalence rates above 10%, implying a homogeneously low prevalence of the LGA and confirming that the LGA does not require PC (Figure 4D).

## DISCUSSION

There is substantial evidence of the burden of schistosomiasis in Nigeria, and efforts to control this disease have been ongoing for over a decade.^18^^–^^22^ Recently, these efforts have shifted toward schistosomiasis EPHP.^3^ Country programs now aim to reduce the proportion of individuals with moderate- and heavy-intensity schistosomiasis infections to less than 1% and decrease the number of tablets required for PC by 50%.^3^ The schistosomiasis PC program in Nigeria has expanded significantly over the years, benefiting from substantial investments from pharmaceutical companies, WHO guidance, and crucial support from governmental and nonprofit organizations.^3^ Since 2014, approximately one fifth of all endemic IUs in Nigeria have started PC, reaching an average of 4–16 million Nigerians annually.^23^ Current projections toward the NTD 2030 Roadmap indicate that approximately 76 million Nigerians across 317 IUs still require PC, whereas approximately 90 IUs might have reached stages of transmission interruption.^23^ Continuing PC in IUs where morbidity has been reduced is counterproductive given limited resources. Therefore, WHO recommends conducting impact assessment surveys after 5 years of effective PC^5^^,^^11^ to redefine endemicity levels, identify hot spots, adjust PC thresholds, determine when to stop or continue PC, and better estimate the resources required.^10^

As many programs are currently planning to conduct these surveys, we present evidence of the sensitivity of some commonly used assessment methodologies that are essential for adjusting PC decisions. This study represents the first impact assessment survey of schistosomiasis in Ondo state, in which 15 sampling sites (schools) were surveyed across each IU. Among the three IUs surveyed, two (Ese-Odo and Ile-Oluiji) exhibited over 90% significant reduction in aggregated prevalence estimates compared with their baseline estimates. Specifically, a prevalence of 1.8%, with only 0.1% harboring heavy intensity, was observed in Ile-Oluiji, a previously hyperendemic IU. These results illustrate the impact of PC, which has been the only intervention for LGAs over the past decade. Similar reports have confirmed significant declines in prevalence estimates after years of PC implementation.^18^^,^^24^^,^^25^

More importantly, in Ese-Odo, recent remapping assessments conducted at the community level among children ages 5–14 years old in 2021 (immediately after the coronavirus disease 2019 [COVID-19] pandemic) revealed three hot spots, particularly in Apoi 1, 4, and 5 wards, with prevalence rates ranging from 22% to 58%.^13^ The sampling methodology, which used one community per subdistrict/ward, was more representative than initial baseline mapping efforts. These findings suggest that the LGA might have cryptic hot spots and may have been misclassified at baseline, supporting our argument for more robust mapping and assessment methodologies. However, our assessment 30 months later in the same location indicated nonendemicity. The discrepancies in prevalence estimates may be attributed to the impact of PC implemented after the mapping exercise or other epidemiological parameters, including differences in the spatiotemporal scales of both assessments (community versus school based and 2021 versus 2024), the approach used in recruiting participants (enrollment of both enrolled and nonenrolled SAC versus only enrolled SAC),^26^^–^^28^ and the participants’ history of involvement in PC programs^29^^,^^30^ and other associated water contact practices that were previously reported elsewhere to be more frequent during the COVID-19 lockdown.^14^^,^^31^ This warrants further investigation in subsequent analyses. Nevertheless, these findings add additional credence to the possibility of cryptic hot spots among nonenrolled SAC who are often missed during regular PC programs typically implemented at schools.^28^ Because community-based studies are more likely to sample children who have never benefited from PC programs and are likely to have higher worm loads, more careful consideration is thus necessary in areas where there are disparities in prevalence estimates between school-based and community-based epidemiological studies.

Furthermore, we observed an increase in prevalence from 3.0% to 5.3% in Irele, with approximately 2.3% of the sampled population harboring high-intensity infections. This LGA was classified as having low endemicity in 2014, and PC was administered biennially until 2021 when outbreaks were reported in some communities.^13^ This prompted the identification of hot spots and led to the reclassification of the LGA for annual PC.^14^ In Nigeria, most baseline epidemiological surveys conducted over a decade ago used sampling methodologies that used only a few sampling sites (typically around five or six) within smaller sampling frames, such as wards or subdistricts, to represent the entire district.^13^ It has been established that the limited number of sampled schools reduces the power to accurately delineate hot spots,^32^ particularly in heterogeneous IUs, where the focal nature of schistosomiasis varies based on ecological and other epidemiological parameters,^33^ or in areas where infections are sparse.^34^

Therefore, we argue that the observed increase in prevalence in Irele, although statistically insignificant, reflects the consequences of underestimating baseline prevalence. This underestimation subsequently influenced the decision to implement PC as the IU was misclassified based on existing evidence. Similar findings have been reported elsewhere, with outbreaks in areas previously considered to have low endemicity.^14^ Thus, it is crucial to emphasize the need to prioritize more robust assessment methodologies over convenience and cost during the formative stages of PC programs.^18^^,^^32^ This includes careful consideration of the scale (district or subdistrict), spread, and number of sampling sites on which the preferred methodologies are built.

WHO has advocated for more robust methodologies to address the limitations of sampling a few schools, such as cluster sampling,^10^ and to capture transmission dynamics across various ecological zones within the IU as seen in the SPPA methodology.^11^ The SPPA methodology introduces a new approach to determining treatment thresholds, such as >10%,^11^ which align with the newly published WHO guidelines for schistosomiasis elimination.^9^ Our comparative analysis of three impact assessment methodologies revealed that the sentinel approach had lower sensitivity in areas where baseline or previous endemicity reports were either consistently low (Ese-Odo) or high (Ile-Oluiji).

However, in Irele, where outbreaks were recently reported after baseline mapping, the sensitivity of the sentinel approach has increased. Conversely, the practical methodology demonstrated higher sensitivity in areas deemed to have low endemicity. As previously emphasized, adjusting PC thresholds is based solely on prevalence estimates obtained from impact assessment surveys. Historically, the most prominent impact assessment evaluations used aggregated estimates at the district level to make treatment decisions using the WHO thresholds of biannual PC when baseline prevalence exceeds 50%, annual PC when prevalence ranges from 10% to 49.9%, and biennial PC when the prevalence falls between 1% and 10%.^5^^,^^34^ However, the latest WHO guidelines have adjusted the prevalence thresholds for PC to >10% and further suggest that decisions should be made at the subdistrict level. Thus, impact assessment methodologies can be modified to align with these guidelines and to make treatment decisions.

## CONCLUSION

In summary, different decisions would have been made if the methodologies had shifted. For instance, in Irele, sentinel and cluster methodologies suggest that the LGA has an aggregate prevalence below 10%, indicating that PC is not required. However, the SPPA approach suggests otherwise as 3 of 15 sampling sites have >10% prevalence.^11^ This reflects the heterogeneity in schistosomiasis transmission in the LGA, indicating the need to optimize the frequency of PC from a biennial mode to an annual mode, particularly in Irele 3, Irele 4, and Omi Iyasan subdistricts. Using Irele, we demonstrate how decisions regarding surveys may have been influenced by the methodology used for assessment. Therefore, it is crucial to emphasize that the percentage changes in prevalence across the methodologies were insignificant, possibly because of the limited number of positive cases. However, this should not be taken seriously as significant differences in prevalence estimates are not currently used to adjust treatment decisions. Nevertheless, our study highlights that the practical assessment methodology is inherently more robust owing to its systematic approach to site selection, larger number of sites, and more restricted eligibility group of children ages 10–14 years old.

One major limitation of this study was the relatively small number of children ages 10–14 years old across a few sampled schools. The SPPA methodology specifies that children who have undergone several rounds of PC are the best target group for monitoring impact, recommending the enrollment of about 30 children ages 10–14 years old. Nigeria has the largest number of out-of-school children in the world, with about 10.5 million children ages 5–14 years old out of school because of economic reasons, the need for labor, early marriage, and poor school quality.^35^ With these high dropout rates, there are likely to be fewer children ages 10–14 years old in school for the SPPA methods. Although an alternative approach would be to sample additional children ages 10–14 years old in the community to meet the target size, this was largely not feasible as the children were often not at home, likely because of work commitments. Therefore, more careful planning for both enrolled and nonenrolled children should be made ahead of mapping or assessment surveys to ensure representative sampling. Additionally, we used a single epidemiological survey to collect data regarding the three different assessment methodologies, and our inferences relied on disaggregated estimations after using data reduction approaches based on the criteria of each assessment methodology. If resources allow, we recommend that independent studies be conducted. Despite these limitations, this study offers valuable insights and provides evidence for adjusting the treatment decisions for LGAs. This study lays the groundwork for further research and exploration of the drivers of transmission in Irele to address the unique challenges that may limit control and elimination efforts.

## Data Availability

The datasets used and/or analyzed during the current study are available at https://zenodo.org/records/11080490.
